# Associations between adherence to 24-h movement guidelines and non-suicidal self-injury in adolescents: separate moderation by sex and mediation by psychological health

**DOI:** 10.3389/fpubh.2026.1840941

**Published:** 2026-07-21

**Authors:** Chenyu Wang, Ye Fan, Xueyan Ren, Yu Zou, Sheng Tan, Yuanquan Yang, Qiqi Yao, Hongsheng Qian

**Affiliations:** 1 School of Physical Education, Wuhan Sports University, Wuhan, China; 2 Department of Sport and Exercise Science, College of Education, Zhejiang University, Hangzhou, China

**Keywords:** movement guidelines, non-suicidal self-injury, physical activity, screen time, sex differences, sleep duration

## Abstract

**Background:**

Adolescent non-suicidal self-injury (NSSI) is a growing public health concern. Adherence to 24-h movement guidelines yields notable health benefits, yet no studies have explored its association with adolescent NSSI. This study examined this association, alongside the moderating effect of sex and mediating role of psychological health (PH).

**Methods:**

A total of 4,205 adolescents from five central Chinese cities were recruited. Self-reported questionnaires assessed sociodemographic characteristics, moderate-to-vigorous physical activity (MVPA), screen time, sleep duration, NSSI, and PH. Logistic regression analyzed associations between 24-h movement guideline adherence and NSSI; sex moderation was tested via interaction terms, and PH mediation via the PROCESS macro.

**Results:**

NSSI prevalence was 35.29%, while only 1.28% adhered to all three guidelines. Compared with adherence to none, meeting more guidelines was associated with progressively lower NSSI odds [1 vs 0: OR = 0.72, 95% CI (0.62, 0.83); 2 vs. 0: OR = 0.44, 95% CI (0.36, 0.54); 3 vs. 0: OR = 0.31, 95% CI (0.15, 0.65)]. However, adherence to three guidelines did not significantly further reduce risk vs. two [OR = 0.72, 95% CI (0.34, 1.49)]. Among equivalent adherence levels, combinations including the sleep guideline were associated with lower NSSI than those without (*p* < 0.05). Isolated MVPA guideline adherence showed no association with NSSI [OR = 1.15, 95% CI (0.74, 1.80)]. Sex significantly moderated the association for two guidelines and the “screen + sleep” combination (*p* < 0.05), with stronger negative associations in girls. PH significantly mediated the relationship.

**Conclusion:**

Promoting adherence to 24-h movement guidelines, particularly the sleep guideline, may help manage adolescent NSSI risks, with emphasis on PH promotion and attention to sex differences.

## 1 Introduction

Non-suicidal self-injury (NSSI) is defined as the deliberate, self-inflicted destruction of body tissue in the absence of suicidal intent (1). Adolescents are particularly vulnerable to NSSI owing to developmental immaturity and challenges in emotion regulation (2). Globally, meta-analytic estimates indicate a pooled lifetime prevalence of 22.0% and a 12-month prevalence of 23.2% among adolescents (2). In China, these figures are slightly higher, at 24.7% (lifetime) and 24.9% (12-month) (3). The high prevalence of NSSI among adolescents not only inflicts direct physical harm but also contributes to a spectrum of adverse outcomes, including mental health disorders and compromised academic performance (4). More critically, NSSI constitutes one of the proximal risk factors for suicide attempts (5, 6), and its frequency is positively associated with an elevated risk of subsequent suicidal behaviors (6, 7). Collectively, NSSI imposes a considerable economic and psychosocial burden on individuals, families, and society. Given the high incidence and long-term deleterious consequences of adolescent NSSI, identifying effective prevention and intervention strategies has emerged as a public health priority (3, 8, 9).

Physical activity (PA), sedentary behavior, and sleep, as modifiable lifestyle factors, show promise in preventing NSSI (10–12). Indeed, recent evidence has shown that sufficient PA (13, 14), limited sedentary behavior (15, 16), and appropriate sleep duration (11, 17) are independently associated with a lower risk of NSSI among adolescents. However, previous research has also reported contrary findings (18). Interestingly, PA, sedentary behavior, and sleep are interdependent behaviors of the 24-h day, collectively termed 24-h movement behaviors (19, 20). Given their compositional nature, any change in one behavior necessarily results in compensatory shifts across the full 24-h movement pattern (19, 20). Therefore, an integrated approach is warranted to examine the association between 24-h movement behaviors and NSSI. In 2016, Canada introduced the world's first 24-h movement guidelines for children and adolescents, which recommend at least 60 min of moderate-to-vigorous physical activity (MVPA) per day, no more than 2 h of screen time, and age-appropriate sleep duration (9 to 11 h for children aged 5–13 and 8 to 10 h for adolescents aged 14–17) (21). This paradigm shift highlights the importance of integrated movement behaviors for health promotion (21, 22) and has been widely adopted by scholars in many countries (23, 24), including China (25).

To date, multiple studies have explored the associations between adherence to 24-h movement guidelines and psychological health (PH) (26–28), along with suicidality and related outcomes (29–31). NSSI has emerged as an increasingly critical public health priority (3, 8, 9), yet no studies to date have assessed the association between 24-h movement guidelines and NSSI, and the existence of such a link remains unconfirmed. Moreover, the prevalence of NSSI among adolescents varies by sex, with higher rates reported in girls (8, 9, 15). Research has also shown sex differences in the association between healthy lifestyle behaviors and NSSI (11, 15). Similar sex variations have been further confirmed in the associations between 24-h movement guidelines and PH (26, 27), as well as suicidality and related outcomes (29–31). However, the extent to which sex moderates the association between adherence to 24-h movement guidelines and NSSI remains unclear.

**Figure 1:**
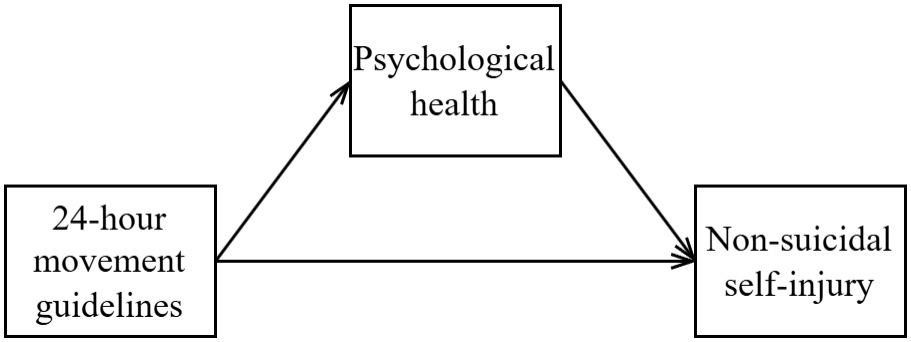
Hypothesized model for the mediating role of PH in the association between adherence to 24-h movement guidelines and NSSI.

PH is defined as a state of mental wellbeing that enables individuals to cope with life stresses, realize personal potential, perform well in study and work, and contribute to the community (32). According to the four-function model of NSSI (33), internal and interpersonal vulnerabilities across emotional, behavioral, and social domains are intertwined, thereby elevating the risk of engaging in NSSI (34, 35). From the perspective of Self-Determination Theory (SDT), chronically unmet basic psychological needs impair PH and increase the propensity to adopt maladaptive compensatory coping strategies such as NSSI (36). Extensive evidence suggests that regular PA, limited screen time, and sufficient sleep are critical pathways for fulfilling basic psychological needs and promoting PH (37–39), which are widely documented as a robust protective factor against adolescent NSSI (8, 40). The 24-h movement framework systematically integrates the daily movement behaviors with health outcomes (21). Adhering to its guidelines is widely regarded as an effective way to improve PH (26, 27). Existing studies have also reported independent inverse associations between 24-h movement behaviors and NSSI (10–13, 15). Synthesizing the above theoretical and empirical evidence, we hypothesize that PH mediates the association between adherence to 24-h movement guidelines and NSSI (Figure 1).

In view of the above theoretical perspectives and existing research gaps, this study aims to: (i) examine the association between adherence to 24-h movement guidelines and NSSI among adolescents; (ii) test whether this association is moderated by sex; and (iii) explore the mediating role of PH in this relationship. The findings are expected to inform the development of interventions targeting 24-h movement behaviors to prevent adolescent NSSI.

## 2 Materials and methods

### 2.1 Participants and procedure

Participants were recruited via convenience cluster sampling from 46 primary and middle schools across five cities in central China between April and June 2025. Previous research indicates that children aged 10 years or older have sufficient reading comprehension skills to complete self-report questionnaires (41). In China, fourth-grade students are typically around 10 years old. Therefore, students in grade 4 and above were eligible for participation.

Written information detailing the study purpose (i.e., assessing demographic characteristics, self-reported PA, screen time, sleep duration, PH, and NSSI), questionnaire procedures, and data use was provided to participants and their guardians, who then furnished written informed consent. They were informed that they could withdraw from the survey at any time. Trained head teachers, school psychological counselors, and graduate students administered and collected the questionnaires on-site. All data were anonymized during collection and analysis. The study was approved by the Ethics Committee of Wuhan Sports University (No. 2025019).

**Figure 2:**
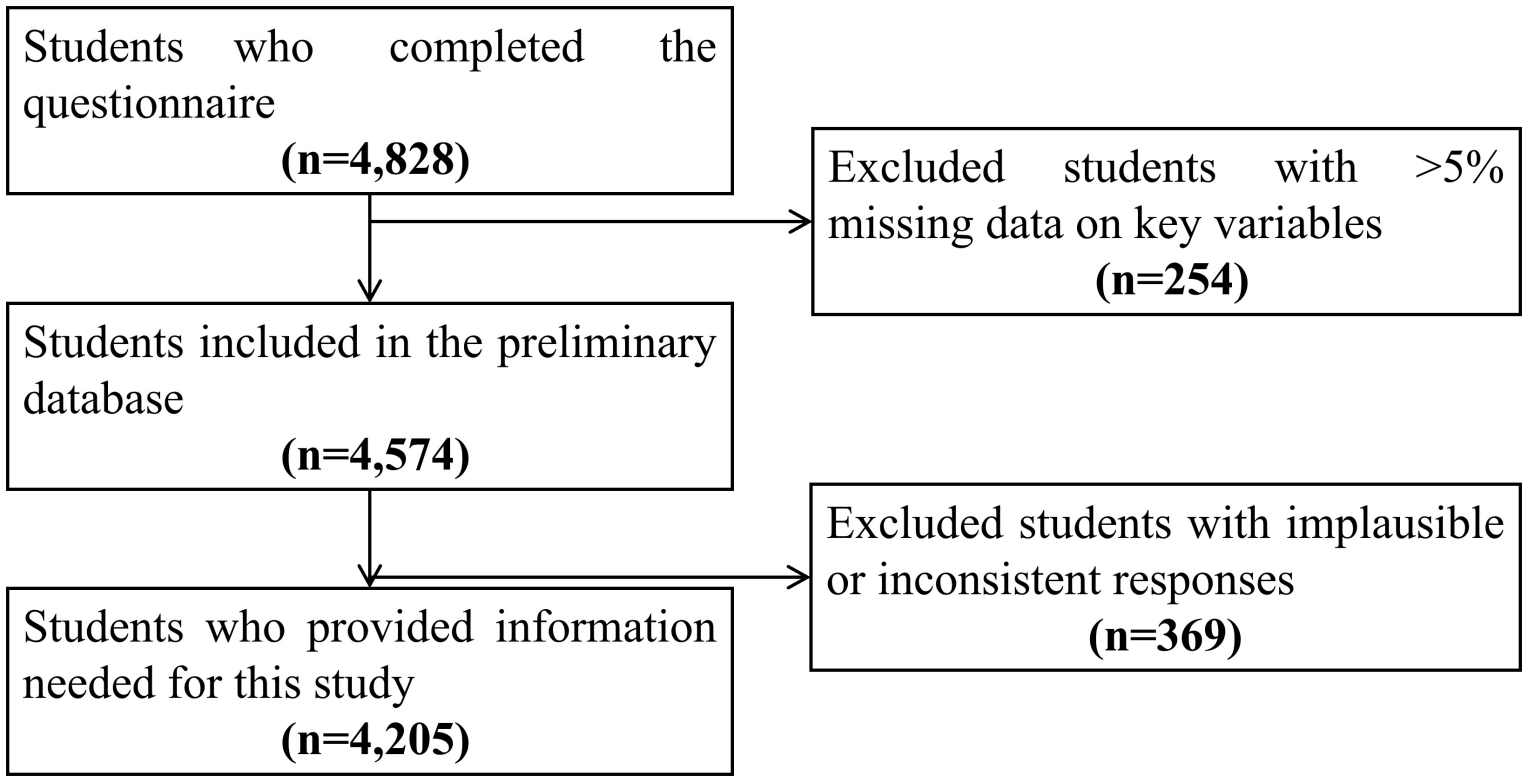
Detailed process used for cleaning invalid and missing data.

A total of 4,828 participants completed the questionnaire. We excluded 623 respondents for the following reasons: (i) missing data on key variables exceeding 5%, or (ii) implausible or inconsistent responses (e.g., PA, screen time, sleep duration, PH or NSSI). The final analytic sample comprised 4,205 students (Figure 2).

### 2.2 Measures

#### 2.2.1 Demographics

Demographic information was gathered as the initial section of the questionnaire, encompassing age, sex, grade, academic score, only-child status, left-behind status, parental education, household income, household-registered residence, and household-registered ethnicity. These variables have been accounted for in statistical analyses to mitigate confounding bias. Thus, during subsequent statistical analysis, these variables (excluding those being examined for interaction effects) were regarded as covariates.

#### 2.2.2 24-h movement behaviors

MVPA was measured using a reliable and valid item derived from the Health Behavior in School-Aged Children (HBSC) survey (42). Participants responded to the following question: “How many days did you engage in moderate-to-vigorous physical activity (MVPA) for at least 60 min in the past week? Responses were coded as: 0 = none, 1 = 1 day, 2 = 2 days, 3 = 3 days, 4 = 4 days, 5 = 5 days, 6 = 6 days, 7 = 7 days”. MVPA was defined as activities resulting in moderate breathlessness and sweating, encompassing daily routines, physical education classes, and additional exercise sessions (43).

Screen time was assessed via a single question adapted from the HBSC survey (42), which quantified time spent on various screen-based behaviors (i.e., TV/movies, video games, and other screen-based activities) in the preceding 7 days. The average daily screen time was computed using a weighted formula to account for differences between school and weekend days, calculated as follows: [(daily screen time on school days × 5) + (daily screen time on weekend days × 2)] ÷ 7.

Sleep duration was quantified via the dedicated sleep duration entry of the Pittsburgh Sleep Quality Index (PSQI) (44), which posed the question: “How many hours did you actually sleep at night in the past month?” The Chinese version of the PSQI has been formally validated and verified for applicability among Chinese children and adolescents (45).

Notably, all the aforementioned items have been widely adopted in empirical research involving Chinese children and adolescents (25, 41, 46, 47). In accordance with the Canadian 24-h Movement Guidelines for Children and Youth (21), adherence to the PA guideline was defined as engaging in ≥60 min of MVPA daily; compliance with the screen time guideline was categorized as ≤ 2 h of daily screen exposure; and meeting the sleep guideline was specified as 9–11 h of nightly sleep for children aged 5–13 years and 8–10 h for adolescents aged 14–17 years, respectively.

#### 2.2.3 Non-suicidal self-injury

NSSI was assessed using the Adolescent Non-Suicidal Self-Injury Assessment Questionnaire (ANSAQ) (48). The behavioral subscale of this questionnaire inquiries about the frequency of NSSI behaviors over the past year, comprising 12 items such as “intentionally pinching oneself”, “intentionally cutting oneself”, and “intentionally burning or scalding oneself”. Each item is rated on a 5-point Likert scale ranging from 0 (never) to 4 (always), yielding a total score between 0 and 48. The subscale has demonstrated strong internal consistency (Cronbach's α = 0.921) and test-retest reliability (r = 0.843) (48), and has been widely used among Chinese children and adolescents (49, 50). In the present study sample, the subscale also exhibited satisfactory internal consistency (Cronbach's α = 0.895). Based on standards from the Diagnostic and Statistical Manual of Mental Disorders (5th Edition, DSM-5), NSSI is clinically characterized by recurrent behaviors occurring on no fewer than 5 days over the past year (51). For the rigor of the present study and with reference to relevant literature (52, 53), a total score of ≥4 was adopted to indicate the presence of NSSI.

#### 2.2.4 Psychological health

PH was measured via the validated and reliable Brief Instrument on Psychological Health of Youths (BIOPHY) (54). This 15-item scale assesses the persistence duration of psychological symptoms, covering three domains: emotional problems, conduct disorder, and social adaptation symptoms. Symptom persistence was scored on a 6-point scale: 1 = ≥3 months, 2 = ≥2 months, 3 = ≥1 month, 4 = ≥2 weeks, 5 = ≥1 week, and 6 = < 1 week or no symptoms. Total scores were derived from the sum of all items, with higher scores indicating better PH status. The BIOPHY shows strong internal consistency (Cronbach's α = 0.928, and split-half coefficient = 0.909) (54), and is widely used in research on Chinese children and adolescents (55, 56). The present sample also showed satisfactory internal consistency (Cronbach's α = 0.922).

### 2.3 Analytical methods and tools

All statistical analyses were carried out with the SPSS software (version 26, IBM Corporation, Armonk, NY, USA). Descriptive statistical methods were employed to present the basic characteristics of the participants. Normally distributed continuous variables (e.g., age) were expressed as mean ± standard deviation (SD), non-normally distributed continuous variables (e.g., PH) as median and interquartile range (IQR), and categorical variables (e.g., paternal education level) as frequencies and percentages. Group differences by sex were assessed using independent samples *t*-tests (for normally distributed continuous variables), Mann-Whitney U tests (for skewed continuous variables), and Chi-square tests (for categorical variables), with statistical significance set at *p* < 0.05.

Logistic regression analyses were performed to examine the associations between adherence to 24-h movement guidelines and NSSI, where NSSI was treated as a dichotomous variable (0 = absence of NSSI, 1 = presence of NSSI). Two separate models were constructed accordingly: (i) evaluating the association between the number of 24-h movement guidelines met (reference group: “0”) and NSSI; and (ii) examining the association between specific combinations of met guidelines (reference group: “none”) and NSSI. To further examine the association between the number of met guidelines and NSSI, we treated the adherence level as an ordinal categorical variable and reported odds ratios for consecutive comparisons (2 vs. 1 and 3 vs. 2). Additionally, we tested the interaction effect of sex on the associations between (i) the number of 24-h movement guidelines met (“0” as the reference group) and (ii) specific guideline combinations (“none” as the reference group) with NSSI. Interaction effects were assessed by including both main effects and the interaction term in the regression models. All models were adjusted for sociodemographic covariates previously specified. Adjusted odds ratios (ORs) and their corresponding 95% confidence intervals (CIs) were reported to quantify the associations.

We explored the mediating role of PH in the associations between two indicators of adherence to 24-h movement guidelines (the number of met guidelines and specific guideline combinations) and NSSI, using Model 4 of the PROCESS macro (version 4.2) for mediation analyses (57). Two distinct sets of analyses were performed: (i) the number of 24-h movement guidelines met as the independent variable (reference group: “0”); and (ii) the specific combinations of 24-h movement guidelines met as the independent variable (reference group: “none”). Demographic variables were included as covariates in all analytical models. The significance of mediating effects was tested via a bootstrapping method with 5,000 resamples and a 95% CI, with significance determined by the absence of zero within the 95% CI.

Little's MCAR test confirmed complete random distribution of missing data (*p* > 0.05) (58). Missing values (overall rate < 5%) were managed via multiple imputation with 20 imputed datasets (59), and cross-dataset results were pooled using Rubin's Rules to generate consolidated estimates, standard errors, and significance levels (60). This approach ensures valid statistical inference by appropriately quantifying missing data-related uncertainty (61).

## 3 Results

### 3.1 Descriptive characteristics of the participants

Descriptive characteristics of participants are presented in Table 1. A total of 4,205 participants (mean age: 12.85 ± 1.33 years) from the primary school (40.78%) and junior middle school (59.22%) were included in this study. There was a roughly balanced sex distribution (42.05% girls) and urban-rural distribution (51.89% urban residents). Most participants were only children (73.08%), lived with at least one parent (60.98% were non-left-behind children), reported a monthly household income between 5,000 and 15,000 CNY (61.31%), and had parents whose highest educational attainment was senior high school or below (76.67%). The mean PH score among participants was 4.96 ± 1.13, with a median (IQR) of 5.33 (4.40–5.80). Regarding adherence to 24-h movement guidelines, 69.44% of participants met at least one recommendation. Among them, 31.56%, 12.01%, and 2.07% of participants met only the screen time guideline, the sleep guideline, and the MVPA guideline, respectively. Only 1.28% of participants met all three guidelines. Regarding the outcome variables, a total of 35.29% of participants were identified as engaging in NSSI. Subsequent inferential analyses revealed significant sex-based disparities across multiple dimensions, specifically in adherence to 24-h movement guidelines, prevalence of NSSI, and scores on the PH scale (*p* < 0.001). Girls exhibited poorer PH and were at higher risk for NSSI, yet demonstrated lower adherence to 24-h movement guidelines (except for the screen time guideline).

**Table 1:** Descriptive characteristics of participants overall and by sex (*n* = 4,205).

Variables		Total sample	Sex		*p*
			Boys	Girls
Age (years old)		12.85 ± 1.33	12.86 ± 1.33	12.84 ± 1.33	0.709
Academic score		76.28 ± 22.81	74.86 ± 23.81	78.22 ± 21.21	< 0.001
Psychological health		4.96 ± 1.13	5.09 ± 1.06	4.78 ± 1.20	< 0.001
Sex	Boys	2,437 (57.95)	2,437 (100)	0	—
	Girls	1,768 (42.05)	0	1,768 (100)	
Ethnicity	Han	4,146 (98.60)	2,397 (98.36)	1,749 (98.93)	0.123
	Minority	59 (1.40)	40 (1.64)	19 (1.07)	
Siblings	Yes	3,073 (73.08)	728 (29.87)	404 (22.85)	< 0.001
	No	1,132 (26.92)	1,709 (70.13)	1,364 (77.15)	
Residence	Urban	2,182 (51.89)	1,261 (51.74)	921 (52.09)	0.783
	Rural	2,023 (48.11)	1,176 (48.26)	847 (47.91)	
Left-behind children	Yes	1,641 (39.02)	983 (40.32)	658 (37.24)	< 0.05
	No	2,564 (60.98)	1,455 (59.68)	1,109 (62.76)	
School level	Primary school	1,715 (40.78)	1,018 (41.77)	697 (39.42)	0.130
	Middle school	2,490 (59.22)	1,419 (58.23)	1,071 (60.58)	
Paternal education level	Junior middle school or below	1,868 (44.42)	1,121 (45.98)	747 (42.25)	< 0.05
	High school or equivalent	1,356 (32.25)	762 (31.25)	595 (33.65)	
	Bachelor or equivalent	905 (21.52)	503 (20.63)	401 (22.68)	
	Master or above	76 (1.81)	52 (2.13)	25 (1.41)	
Household income	≤ 5,000 CNY/mouth	899 (21.38)	495 (20.31)	404 (22.85)	< 0.05
	5,000–10,000 CNY/mouth	1,735 (41.26)	1,008 (41.36)	726 (41.06)	
	10,000–15,000 CNY/mouth	843 (20.05)	485 (19.90)	359 (20.31)	
	15,000–20,000 CNY/mouth	307 (7.30)	172 (7.06)	135 (7.64)	
	≥20,000 CNY/mouth	421 (10.01)	277 (11.37)	144 (8.14)	
Adherence to the 24-h movement guidelines	None	1,285 (30.56)	682 (27.97)	603 (34.13)	< 0.001
	One	1,919 (45.64)	1,137 (46.64)	782 (44.26)	
	Two	947 (22.52)	579 (23.75)	368 (20.83)	
	Three	54 (1.28)	40 (1.64)	14 (0.79)	
	MVPA only	87 (2.07)	63 (2.59)	24 (1.36)	< 0.001
	Screen only	1,327 (31.56)	719 (29.50)	608 (34.39)	
	Sleep only	505 (12.01)	354 (14.53)	151 (8.54)	
	MVPA + screen	80 (1.90)	56 (2.30)	24 (1.36)	
	MVPA + sleep	34 (0.81)	23 (0.94)	11 (0.62)	
	Screen + sleep	833 (19.81)	500 (20.52)	333 (18.83)	
Non-suicidal self-injury	Yes	1,484 (35.29)	758 (31.10)	727 (41.12)	< 0.001
	No	2,721 (64.71)	1,679 (68.90)	1,041 (58.88)	

### 3.2 Association between adherence to 24-h movement guidelines and NSSI

**Figure 3:**
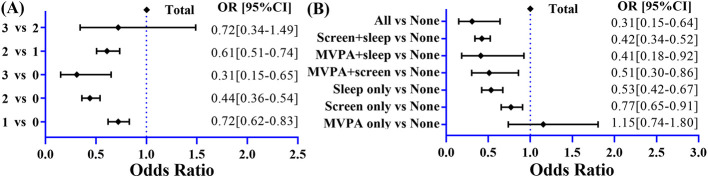
Associations between adherence to 24-h movement guidelines and NSSI. **(A)** ORs (95% CIs) for NSSI by number of met guidelines. **(B)** ORs (95% CIs) for NSSI by guideline combinations. OR, odds ratio; CI, confidence interval.

Figure 3 presents the associations between adherence to 24-h movement guidelines and NSSI. As shown in Figure 3A, compared with meeting none of the guidelines, adhering to a greater number of guidelines was significantly associated with a lower prevalence of NSSI [OR for 1 vs. 0: 0.72 95% CI (0.62, 0.83); for 2 vs. 0: 0.44 95% CI (0.36, 0.54); for 3 vs. 0: 0.31 95% CI (0.15, 0.65)], indicating a significant inverse dose-response relationship. Specifically, meeting two guidelines was associated with a significantly lower likelihood of NSSI than meeting only one [OR = 0.61, 95% CI (0.51, 0.74)]. However, meeting all three guidelines did not confer a further statistically significant reduction in NSSI prevalence compared with meeting two [OR = 0.72, 95% CI (0.34, 1.49)], suggesting diminishing returns beyond two recommendations.

As shown in Figure 3B, compared with meeting none of the guidelines, adherence to any combination of guidelines (except MVPA only) was significantly associated with lower odds of NSSI. Among participants meeting only one guideline, those adhering to the sleep guideline showed the strongest protective association [OR = 0.53, 95% CI (0.42, 0.67)]. Among two guideline combinations, both “screen + sleep” [OR = 0.42, 95% CI (0.34, 0.52)] and “MVPA + sleep” [OR = 0.41, 95% CI (0.18, 0.92)] were similarly protective and more strongly associated with reduced NSSI risk than the “MVPA + screen” combination [OR = 0.51, 95% CI (0.30, 0.86)]. Adherence to all three guidelines was associated with the lowest odds of NSSI compared with meeting none [OR = 0.31, 95% CI (0.15, 0.64)], indicating the strongest inverse association with NSSI.

### 3.3 Moderating role of sex in the association between adherence to 24-h movement guidelines and NSSI

**Figure 4:**
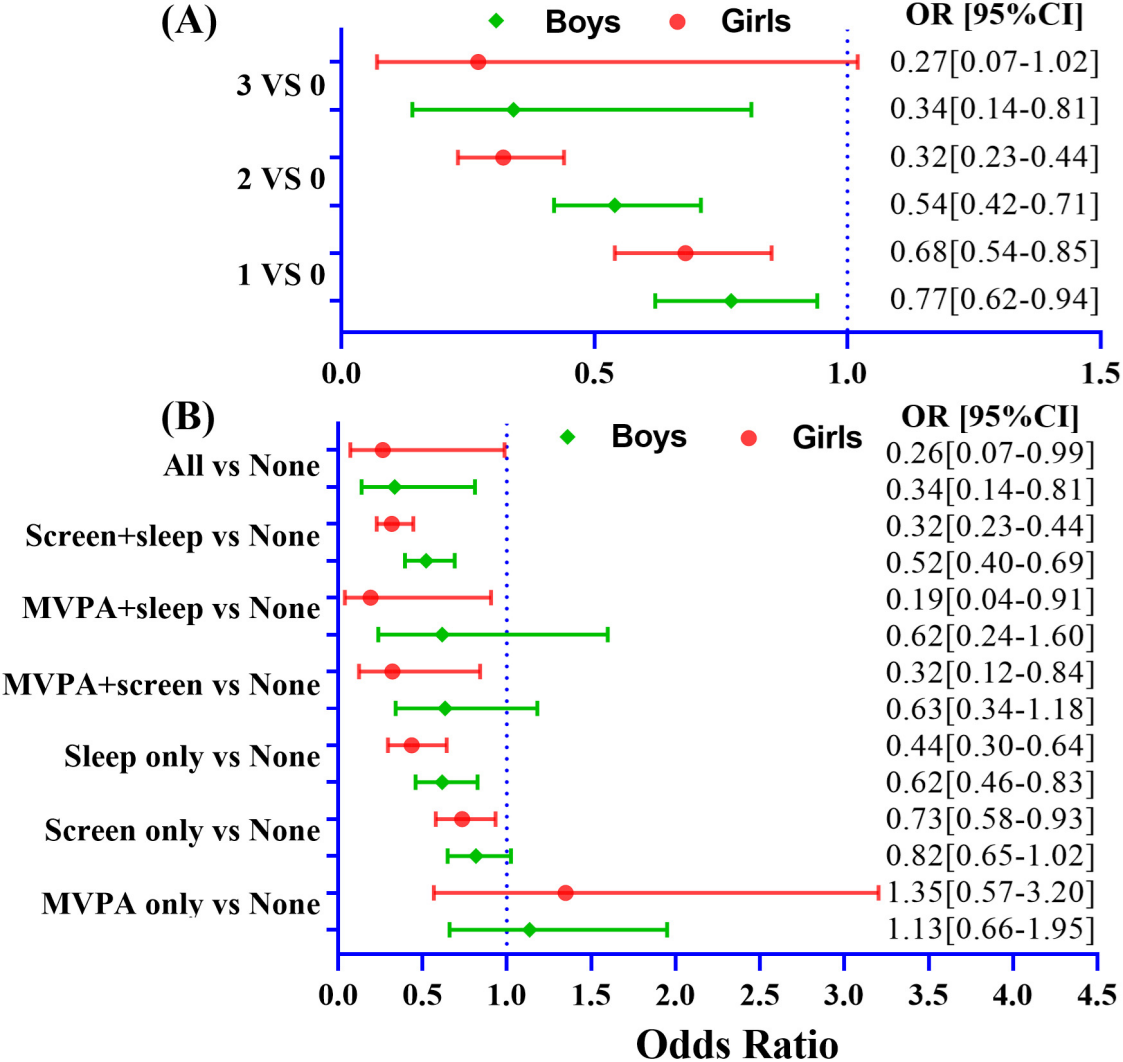
Associations between adherence to 24-h movement guidelines and NSSI by sex. **(A)** ORs (95% CIs) for NSSI by number of met guidelines. **(B)** ORs (95% CIs) for NSSI by guideline combinations. OR, odds ratio; CI, confidence interval.

To examine whether sex moderates the association between the number of 24-h movement guidelines met and NSSI, we fitted a logistic regression model including an interaction term between the number of guidelines met and sex. The results showed a significant overall interaction effect [Wald χ^2^ (3) = 9.47, *p* < 0.05], indicating that the association between the number of 24-h movement guidelines met and NSSI differed by sex. Specifically, for those adhering to two of the 24-h movement guidelines, the inverse association with NSSI was markedly weaker in boys relative to girls [OR = 1.76, 95% CI (1.20, 2.58), *p* < 0.01]. In contrast, no significant sex differences were observed in the inverse associations for adhering to one (*p* = 0.216) or three (*p* = 0.642) guidelines. To further verify and refine this finding, we conducted sex-stratified analyses of the association between guideline adherence and NSSI (Figure 4A). The results revealed that the inverse associations between any level of guideline adherence and NSSI were stronger among girls (i.e., ORs for adhering to one guideline: 0.68 vs. 0.77), with girls exhibiting a markedly lower OR (0.32) for NSSI than boys (0.54) when meeting two guidelines. This further confirms the robustness of the interaction effect and suggests that girls show a stronger inverse association between guideline adherence and lower NSSI, particularly when meeting two guidelines.

Similarly, we further examined whether sex exerts a moderating effect on the association between combination of met components of the 24-h movement guidelines and NSSI. The results revealed no significant overall interaction effect [Wald χ^2^ (7) = 11.36, *p* = 0.124]. However, a significant sex difference was observed in the association between adherence to the “screen + sleep” guidelines combination and NSSI [OR = 1.69, 95% CI (1.13, 2.52), *p* < 0.05], whereas no significant sex differences emerged for other guideline combinations (*p* > 0.05). Stratified analyses (Figure 4B) further showed that the association between adherence to the “screen + sleep” guidelines and NSSI was stronger in girls [OR = 0.32, 95% CI (0.23, 0.44)] than in boys [OR = 0.52, 95% CI (0.40, 0.69)], suggesting a stronger inverse association among girls.

### 3.4 Mediating role of PH in the association between adherence to 24-h movement guidelines and NSSI

An exploratory factor analysis was performed to evaluate common method bias. The first principal component accounted for 20.83% of the variance (with five factors exceeding an eigenvalue of 1), which is below the 40% criterion (62), suggesting minimal common method bias.

**Figure 5:**
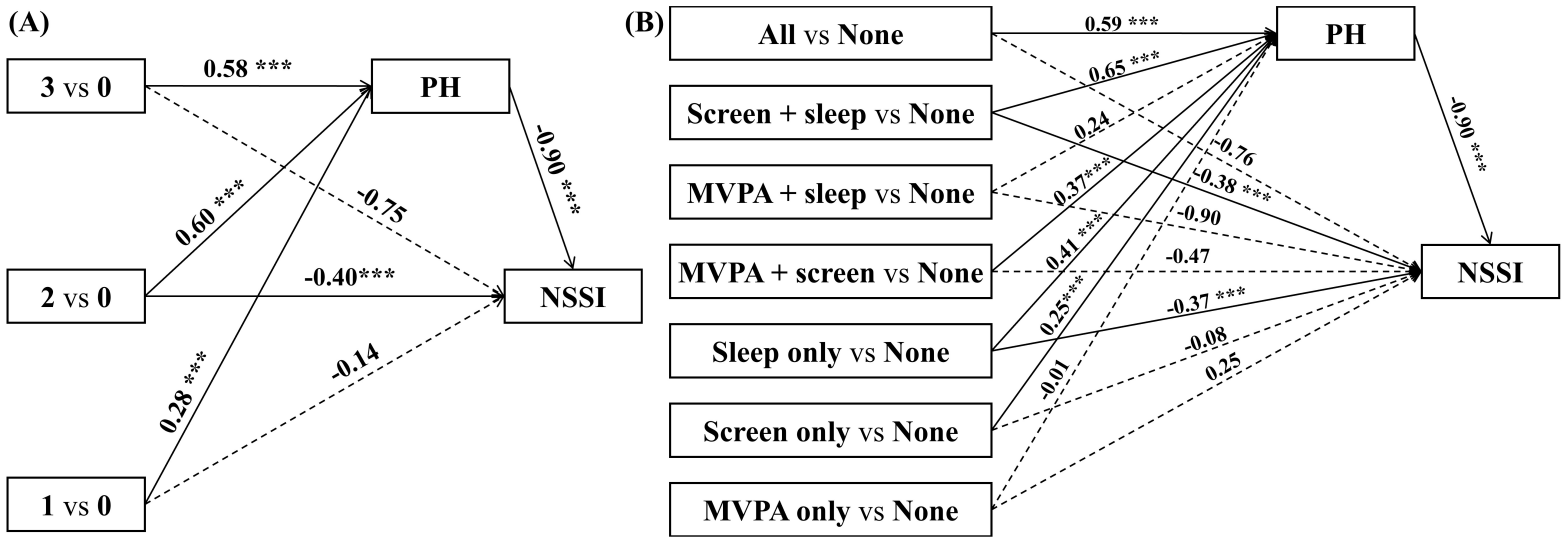
Mediating role of PH in the association between adherence to 24-h movement guidelines and NSSI. **(A)** Mediation effects by the number of met guidelines. **(B)** Mediation effects by specific guideline adherence combinations. Solid lines indicate significant associations; dotted lines represent non-significant paths. ^***^p < 0.001.

Figure 5A illustrates the mediating role of PH in the association between the number of 24-h movement guidelines met and NSSI. In this model, adherence to a greater number of guidelines was significantly and positively associated with higher levels of PH (1 vs. 0: β = 0.28, *p* < 0.001; 2 vs. 0: β = 0.60, *p* < 0.001; 3 vs. 0: β = 0.58, *p* < 0.001). PH showed a significant negative association with NSSI (β = −0.90, *p* < 0.001). Regarding direct effects, only adherence to two guidelines was significantly inversely associated with NSSI (β = −0.40, *p* < 0.001), whereas adherence to one (*p* = 0.114) or three (*p* = 0.059) guidelines did not show statistically significant direct associations with NSSI.

Further examination of direct and indirect effects (Table 2) revealed that the indirect effects of adhering to two [β = −0.54, 95% CI (−0.64, −0.44)] and three [β = −0.52, 95% CI (−0.74, −0.31)] of the 24-h movement guidelines were comparable in magnitude and substantially larger than those of adhering to only one guideline [β = −0.25, 95% CI (−0.33, −0.18)]. These findings indicate that PH is a key mediator in the association between guideline adherence and NSSI. Notably, adherence to all three guidelines showed a non-significant direct effect on NSSI [β = −0.75, 95% CI (−1.53, 0.03)], with the wide CI indicating low precision.

**Table 2:** Direct and indirect effects of adherence to the number of 24-h movement guidelines on NSSI.

Model	Path	Effect	*SE*	LLCI	ULCI
Direct effect	1 → NSSI	−0.14	0.09	−0.30	0.03
	2 → NSSI	−0.40	0.12	−0.62	−0.17
	3 → NSSI	−0.75	0.40	−1.53	0.03
Indirect effect	1 → PH → NSSI	−0.25	0.04	−0.33	−0.18
	2 → PH → NSSI	−0.54	0.05	−0.64	−0.44
	3 → PH → NSSI	−0.52	0.11	−0.74	−0.31

Not meeting any guidelines served as the reference group for all models.

Figure 5B illustrates the mediating role of PH in the association between specific combinations of 24-h movement guideline adherence an NSSI. In this model, PH also exhibited a significant negative association with NSSI (β = −0.90, *p* < 0.001). Adherence to most guideline combinations (with the exceptions of MVPA only and “MVPA + sleep”) was significantly positively associated with higher PH levels (*p* < 0.001). Regarding direct effects, only adherence to sleep alone (β = −0.37, *p* < 0.001) and “screen + sleep” (β = −0.38, *p* < 0.001) were significantly negatively associated with NSSI, and all other direct paths were non-significant (*p* > 0.05).

Further examination of direct and indirect effects (Table 3) revealed that the indirect effects of adhering to the “screen + sleep” [β = −0.58, 95% CI (−0.68, −0.48)] and the all three guidelines [β = −0.53, 95% CI (−0.75, −0.31)] were comparable in magnitude and substantially larger than those of other guideline combinations. Among those adhering to only one guideline, adherence to sleep only exhibited the largest significant direct [β = −0.37, 95% CI (−0.63, −0.11)] and indirect [β = −0.38, 95% CI (−0.49, −0.26)] effects. In contrast, adherence to MVPA only showed no significant direct [β = 0.25, 95% CI (−0.25, 0.76)] or indirect [β = 0.01, 95% CI (−0.27, 0.28)] effects. These findings further confirm that PH is a key mediator in the protective association between guideline adherence and NSSI, and they highlight the particularly strong inverse association for adherence to sleep guideline. Notably, adherence to “MVPA + sleep” [β = −0.90, 95% CI (−1.79, 0)] and all three [β = −0.76, 95% CI (−1.54, 0.02)] guidelines showed non-significant but negative direct effects on NSSI, with wide CIs reflecting considerable uncertainty.

**Table 3:** Direct and indirect effects of adherence combinations to the 24-h movement guidelines on NSSI.

Model	Path	Effect	*SE*	LLCI	ULCI
Direct effect	MVPA only → NSSI	0.25	0.26	−0.25	0.76
	Screen only → NSSI	−0.08	0.09	−0.26	0.11
	Sleep only → NSSI	−0.37	0.13	−0.63	−0.11
	MVPA + screen → NSSI	−0.47	0.30	−1.05	0.11
	MVPA + sleep → NSSI	−0.90	0.46	−1.79	0
	Screen + sleep → NSSI	−0.38	0.12	−0.62	−0.14
	All → NSSI	−0.76	0.40	−1.54	0.02
Indirect effect	MVPA only → PH → NSSI	0.01	0.14	−0.27	0.28
	Screen only → PH → NSSI	−0.23	0.04	−0.31	−0.14
	Sleep only → PH → NSSI	−0.38	0.06	−0.49	−0.26
	MVPA + screen → PH → NSSI	−0.33	0.12	−0.57	−0.09
	MVPA + sleep → PH → NSSI	−0.22	0.18	−0.56	0.13
	Screen + sleep → PH → NSSI	−0.58	0.05	−0.68	−0.48
	All → PH → NSSI	−0.53	0.11	−0.75	−0.31

Not meeting any guidelines served as the reference group for all models.

## 4 Discussion

### 4.1 Main findings

The main findings of this study are as follows: (i) adherence to all three of the 24-h movement guidelines is low among adolescents, while the prevalence of NSSI is relatively high, with significant differences observed by sex; (ii) an inverse dose-response relationship exists between guideline adherence and NSSI odds. However, this protective effect plateaus after meeting any two components, suggesting a potential diminishing marginal return; (i) adherence to the sleep guideline shows the strongest inverse association with NSSI, followed by the screen guideline, whereas meeting the MVPA guideline alone is not significantly associated with NSSI odds; (ii) sex may moderate the association between guideline adherence and NSSI, with stronger protective association observed among girls; (iii) PH significantly mediates the association between adherence to 24-h movement guidelines and NSSI. These findings confirm the inverse dose-response relationship and diminishing marginal effect between adherence to 24-h movement guidelines and NSSI. The findings may provide new ideas for developing personalized prevention strategies targeting adolescent NSSI, with sleep guideline adherence worthy of particular attention.

### 4.2 Adherence to 24-h movement guidelines as a modifiable protective factor against adolescent NSSI

To our knowledge, this is the first study to examine the association between adherence to 24-h movement guidelines and NSSI in adolescents. In this sample, only 1.28% of adolescents met the overall guidelines. This finding is consistent with prior studies among Chinese adolescents (46, 47), though the prevalence appears likely lower than estimates reported in some other countries (39). Previous evidence indicated markedly lower adherence to 24-h movement guidelines among primary and secondary school students than among older adolescents, possibly due to heightened academic pressures and less mature self-regulatory capacity (28, 63). In terms of meeting individual movement guidelines, 2.07%, 31.56%, and 12.01% of participants met the MVPA, screen time, and sleep duration guidelines, respectively. Notably, adherence to any combination of guidelines that included MVPA was below 2%. These findings align with global trends showing that the majority of adolescents fail to meet current PA recommendations (64), underscoring the urgent need for targeted strategies to promote PA in this population. Furthermore, the NSSI prevalence among adolescents in the present study was 35.29%, which aligns with the results of Tang et al. (17) and Liu et al. (65), but is substantially higher than the two meta-analytic estimates: 24.9% for the Chinese adolescent population (3) and 17.7% across global adolescent samples (9). Previous research has indicated that this discrepancy is associated with academic pressure, family dynamics, peer interactions, environmental factors, and individual lifestyles among Chinese adolescents (3, 8, 9, 11, 12). In summary, this highlights the urgency of addressing adolescent NSSI, underscoring the necessity of implementing targeted preventive interventions.

A key finding of the present study is that adherence to a greater number of the 24-h movement guidelines was associated with incrementally lower odds of NSSI, consistent with an inverse dose-response relationship (i.e., odds: 0 > 1 > 2 > 3). Previous studies have shown that sufficient PA (13, 14), limited sedentary behavior (15, 16), and appropriate sleep duration (11, 17), are each linked to reduced NSSI risk in adolescents. Given their individual benefits, these behaviors may exert cumulative protective effects against NSSI when combined (11). This hypothesis is partially supported by our findings, which align with previous evidence linking greater guideline adherence to better mental health (39) and lower odds of suicidal ideation and suicide attempts (29). Although a dose-response trend was evident, the incremental benefit of meeting a third guideline component was small and statistically non-significant, suggesting possible diminishing marginal returns. The observed dose-response relationship with diminishing marginal returns suggests that meeting more of the 24-h movement guidelines remains the optimal strategy for maximizing protective benefits against NSSI. Nevertheless, even partial adherence to these guidelines can yield substantial risk reduction, supporting the development of targeted, cost-effective interventions that prioritize high-leverage components when resources are limited. Notably, the small number of participants adhering to all three guidelines may have contributed to imprecise effect estimates (evidenced by wide CIs), warranting cautious interpretation and the need for confirmation in larger observational and experimental studies.

In addition, among combinations adhering to the same number of guidelines, those that included meeting the sleep guideline demonstrated the strongest protective association with NSSI. This suggests that adherence to the sleep guideline may play a potential leading role in lowering NSSI odds, which is consistent with the findings of Khazaie et al. (10). Biologically, adequate sleep stabilizes neuroendocrine balance, preventing melatonin and cortisol disruptions, which reduces emotional dysregulation and impulsivity (10). Moreover, normal sleep improves prefrontal cortex functioning, enhancing the brain's ability to inhibit responses to negative emotional stimuli, thereby lowering self-harm risk (66). Sociologically, adequate sleep improves cognitive flexibility and interrupts negative thinking (67). Furthermore, regular sleep enhances adolescents' motivation for social participation and their social adaptability, thereby reducing the likelihood of NSSI (10, 68). Given the critical role of sleep, adherence to sleep guidelines should be prioritized in future interventions targeting adolescents.

Another key finding of this study is that both adherence to the screen time guideline alone and its inclusion in broader behavioral combinations are significantly associated with lower NSSI risk among adolescents, a result consistent with prior research (11, 15). The protective role of limiting screen time can be understood from two complementary perspectives. First, reduced screen exposure decreases adolescents' contact with harmful online content, thereby lowering the risk of imitative NSSI (15) and attenuating the co-occurrence of internet addiction and NSSI (69). Moreover, less screen dependence fosters face-to-face interaction and real-world engagement, strengthening social bonds and alleviating isolation-related distress, thereby reducing reliance on self-injury for emotional regulation (15). Importantly, combinations that include the screen time guideline confer even stronger protection. When combined with adequate sleep, it promotes circadian rhythm stability and disrupts the detrimental cascade from excessive screen time to sleep loss and subsequent emotional dysregulation (10). When combined with MVPA, it promotes offline engagement, improving neuroendocrine balance through the regulation of serotonin and dopamine (67) and reinforcing social support via in-person interactions (68), thereby reducing NSSI risk through dual biological and social pathways.

Unfortunately, adhering to the MVPA guideline alone showed no significant protective association with NSSI, which contradicts previous findings that PA reduces NSSI risk (12–14). Given that concurrent adherence to multiple components of the 24-h movement guidelines is significantly associated with decreased NSSI risk, this suggests that the association between MVPA and NSSI may depend on co-occurring movement behaviors, such as sleep duration and screen time, rather than MVPA in isolation (10). Another potential explanation is that this study only assessed MVPA duration and did not account for the context or type of MVPA. Prior research has indicated that voluntary and team-based exercise is more likely to mitigate NSSI risk, whereas mandatory and individual exercise may have a weaker association with NSSI reduction (70). Furthermore, some studies have highlighted that the impact of PA on NSSI requires specific mediating factors (e.g., interpersonal connections) to exert its protective effect (10, 49, 68, 71).

Collectively, the findings indicate a trend whereby meeting a greater number of the 24-h movement guidelines is linked to reduced odds of NSSI, aligning with the 24-h movement framework that frames daily behaviors as an interconnected compositional system instead of separate factors (21). In instances where full adherence is unattainable, compliance with any two of the guidelines still confers considerable protective benefits. Notably, the association between guideline adherence and NSSI appears to be primarily driven by sleep duration, followed by screen time, and then MVPA, highlighting distinct protective magnitudes for each component in this integrated framework (21). This underscores the paramount importance of promoting sufficient sleep as potential strategies to mitigate NSSI risk. Nonetheless, given that this is the inaugural study exploring the association between 24-h movement guidelines and NSSI, and considering the limited number of participants who adhered to all three guidelines, these findings should be interpreted with caution and warrant confirmation in larger cohorts.

### 4.3 Sex differences in the association between adherence to 24-h movement guidelines and NSSI

Aligned with existing evidence, this study confirms that adolescent girls exhibit low adherence to the majority of 24-h movement guidelines (28, 41) and a relatively high prevalence of NSSI (3, 8, 9). However, adherence to 24-h movement guidelines exerts a more marked protective effect against NSSI in girls, which aligns with previous evidence linking guideline compliance to lower risks of depressive symptoms and suicide-related behaviors (i.e., suicidal ideation, planning, and attempts) (27, 30). This finding indicates that unhealthy movement behaviors may exert a more pronounced adverse influence on the occurrence of NSSI among girls (11).

Specifically, compliance with two of the 24-h movement guideline recommendations correlated with a more robust protective association with NSSI among girls. In contrast, no significant sex-based differences were observed in the association between NSSI and adherence to any other quantity of guideline components. Two potential factors may underlie this pattern. On one hand, previous studies have identified that Chinese boys are more inclined to meet the MVPA guideline, whereas girls tend to adhere more closely to the screen time recommendation (46). The present study observed an analogous pattern. Meanwhile, our study observed that boys were more likely to comply with MVPA-inclusive combinations (e.g., “MVPA + screen” or “MVPA + sleep”), while girls predominantly adhered to the “screen + sleep” combination. As previously noted, the “screen + sleep” combination demonstrated a stronger protective association with NSSI. Therefore, sex-specific preferences in movement behavior profiles may constitute a key contributor to this observed phenomenon. On the other hand, internalizing problems such as depression, anxiety and emotional dysregulation exert a stronger influence on NSSI among girls, while externalizing problems including impulsivity, aggressive behaviors and substance use are more closely linked to NSSI in boys (8, 9). Prior studies have demonstrated that concurrent adherence to screen and sleep guidelines offers greater protection against internalizing problems such as depression and anxiety (27, 31). This may also explain the lower incidence of NSSI observed in girls who adhere to these two guidelines.

No significant sex differences were observed in the association between NSSI and adherence to only one or to all three of the 24-h movement guidelines. Meeting just one guideline was associated with exposure to multiple concurrent NSSI risk factors in both boys and girls. These overlapping risks may obscure sex-related effects. Conversely, adherence to all three guidelines is associated with the strongest protective effect against NSSI and other mental health problems (27, 30), which may mask underlying sex differences due to the magnitude of this overall benefit. It should be noted that the prevalence of adolescents meeting all three guidelines is extremely low (46, 47). This limited adherence may have reduced the statistical power of subgroup analyses, potentially obscuring sex differences.

A further key finding is that adherence to the combined screen time and sleep guidelines is linked to markedly lower NSSI risk in adolescent girls relative to boys. This sex difference likely stems from distinct neurobiological sensitivities, behavioral patterns, and risk exposure profiles (26, 27, 67). Neurobiologically, pubertal hormonal fluctuations and sex-specific prefrontal cortical development increase girls' susceptibility to emotional dysregulation due to social media-related sleep disruption, an effect that is mitigated by adherence to the screen time and sleep guidelines (27, 30). Behaviorally, girls' social media use directly impairs sleep, so reduced screen time amplifies protective benefits, whereas boys' predominantly gaming-focused screen use has weaker sleep links, limiting such effects (26, 27, 31). In terms of risk exposure, girls have a higher baseline NSSI prevalence, with their internalizing problems more strongly linked to screen time and sleep disruption. Adherence to the guidelines is correlated with more noticeable declines in NSSI risk for girls. In contrast, boys' NSSI is associated with broader stressors and externalizing problems (27, 67). Collectively, these mechanisms explain the observed sex difference across these associations.

Notably, although no significant sex differences were observed in the associations between NSSI and adherence to guidelines other than the “screen + sleep” combination, ORs for most individual guidelines (except MVPA alone) and their combinations were consistently lower in girls than in boys, suggesting stronger protective effects in girls. Moreover, among boys, adherence to MVPA, screen time, or the combinations of “MVPA + screen” or “MVPA + sleep” was not significantly associated with reduced NSSI risk. These findings further support the notion that adolescent girls may be more sensitive to the association between adherence to 24-h movement guidelines and NSSI risk. Specifically, adherence to the sleep and screen time guidelines conferred stronger associations with lower NSSI risk in girls compared to boys, whereas MVPA showed no significant correlation in both sexes. Additionally, the low adherence rates to MVPA and its combinations in this sample may have resulted in insufficient statistical power, potentially obscuring true sex differences in the association between MVPA-related behaviors and NSSI.

In summary, the sex-specific findings of this study provide actionable directions for the targeted prevention of NSSI in adolescents. For girls, promoting adherence to 24-h movement guidelines, particularly the combined sleep and screen time recommendations, is likely to yield substantial protective benefits against NSSI. For boys, while adherence to sleep and screen time guidelines remains important, adherence to all three guidelines including MVPA may be necessary to achieve meaningful reductions in NSSI risk. That said, several limitations warrant caution when interpreting these findings. Low adherence rates to certain guidelines, especially MVPA, and the lack of differentiation by MVPA context or type, screen-based behaviors such as social media vs. gaming, and sleep quality may have obscured more nuanced sex differences. Future research incorporating more granular behavioral measures and larger subsamples is needed to elucidate the underlying mechanisms driving these sex-specific associations.

### 4.4 PH as an underlying pathway between adherence to 24-h movement guidelines and NSSI

A further key contribution is the first empirical validation that PH significantly mediates the association between adherence to 24-h movement guidelines and adolescent NSSI. Extensive evidence shows that adherence to 24-h movement guidelines improve PH (26, 38, 39), and favorable PH reduces NSSI risk through multiple pathways, including emotion regulation, self-efficacy, and social support (1, 3, 8, 12, 54). These findings highlight PH as a critical bridge connecting 24-h movement behaviors to NSSI prevention. Consistent with SDT (36), health movement behaviors fulfill adolescents' basic psychological needs to build protective psychological resources against NSSI (1, 49, 71). Meanwhile, this study extends prior isolated-behavior research to the integrated 24-h movement framework (21, 54, 72).

In terms of the number of guidelines met, the mediating effect of PH on the association between 24-h movement behaviors and NSSI was significantly stronger when adolescents met two or three guidelines compared with meeting only one. This result strongly supports the core tenet of the 24-h movement framework that multiple health behaviors have a cumulative effect on PH (21, 39). When only one guideline is met, the limited psychological benefits conferred by that single behavior may be offset by the compounded risks arising from noncompliance with the other two. For instance, the psychological benefits conferred by endorphin release during PA can be counteracted by social isolation resulting from excessive screen time and neurotransmitter imbalance caused by insufficient sleep (38, 39). As the number of guidelines met increases, risk exposure diminishes and synergistic compliance with multiple behaviors continuously accumulates psychological protective resources (11, 39), thereby laying a solid foundation for the full exertion of the mediating effect. Notably, the direct effect of full guideline adherence on NSSI was marginally significant [95% CI (−1.532, 0.027)], indicating that its influence on NSSI is primarily mediated by PH, while other unexplored mediators remain to be investigated.

The magnitude of the mediating effect of PH varies across different combinations of the 24-h movement guidelines that participants met. This further elucidates that those specific behavioral patterns form a personalized link with the psychological risk factors for NSSI. Combinations involving compliance with both screen time and sleep duration guidelines (including adherence to all three guidelines) demonstrated a significantly more robust mediating effect compared to combinations without these two core behaviors, consistent with evidence that adherence to screen time and sleep guidelines serves as the strongest predictor of PH (39). Adolescents with NSSI universally display psychological features like emotion dysregulation and compromised inhibitory control (1, 3, 8). Adherence to screen time guidelines diminishes cognitive distortions induced by exposure to detrimental content and social comparison (73), whereas adherence to sleep guidelines restores prefrontal cortical function, optimizes emotion regulation and boosts executive function (74, 75). Such behavioral synergy directly targets and alleviates these core psychological deficits (38, 39), consequently amplifying the mediating effect. Adherence to the MVPA guideline alone showed non-significant direct and indirect pathways to NSSI. Current findings on the impact of MVPA on PH and NSSI remain controversial (10, 38, 39), potentially due to self-report bias and study design limitations (39), which warrant further investigation. Furthermore, adherence to the “MVPA + sleep” combination exhibited non-significant direct and indirect pathways to NSSI, yet the direct pathway showed a large effect size (β = −0.895) and marginal significance [95% CI (−1.793, 0.003)]. This observation indicates that this combination may exert a potential direct impact on NSSI, yet partial effects have been absorbed by PH mediation with potential sample size-related interference. Adherence to all three guidelines did not significantly enhance the mediating effect on NSSI compared with the “screen + sleep” guideline combination, indicating that adherence to these two guidelines already provides essentially all key psychological resources for NSSI prevention, with MVPA serving a more auxiliary reinforcing role (39). This further supports that the mediating effect of PH is driven by synergistic interactions among core 24-h movement behaviors, not by the mere addition of individual behaviors.

Collectively, our findings suggest that PH may partially account for the inverse association between adherence to 24-h movement guidelines and NSSI among adolescents. This highlights the potential value of promoting key movement behaviors, particularly sufficient sleep and limited screen time, to foster PH and inform NSSI prevention under the integrated 24-h movement framework.

### 4.5 Limitations and strengths

This study has several inherent limitations. First, the cross-sectional design precludes causal inference, and longitudinal or experimental studies are needed to clarify the directional relationship between variables. Second, self-reported data may be subject to recall bias and social desirability bias, which objective measures could help mitigate. Third, although participants were recruited from multiple cities to reflect urban–rural differences and demographic diversity, the sampling strategy did not employ a rigorous stratified probability design, limiting the generalizability of the findings. Thus, the generalizability of the findings needs to be interpreted with caution. Finally, although all assessment tools employed in this study have undergone extensive validation, the study failed to distinguish among MVPA types (aerobic vs. resistance exercise), MVPA contexts (team-based vs. individual exercise), screen time categories (educational vs. recreational use), and circadian rhythms (regular vs. disrupted sleep). These aspects call for more detailed exploration in subsequent studies.

Nevertheless, the study also has notable strengths. Sex, age, only-child status, left-behind status, urban-rural residence, school level, academic performance, parental education, and monthly household income were included as covariates in the analyses, enhancing the robustness of the results. Additionally, this is among the first studies to examine the association between adherence to 24-h movement guidelines and NSSI in adolescents, and the first to explore this relationship by gender. It is also the first to confirm the mediating role of PH in the association between adherence to 24-h movement guidelines and NSSI. These contributions advance the understanding of how 24-h movement behaviors influence NSSI risk and inform the development of targeted NSSI prevention strategies for adolescents.

## 5 Conclusion

The present study confirms a negative dose-response association featuring diminishing marginal returns between adherence to 24-h movement guidelines and adolescent NSSI, identifying adherence to the sleep guideline as the primary protective factor, followed by compliance with the screen time guideline. Methodologically, this study advances existing literature by comprehensively evaluating both the quantitative indicators and specific behavioral characteristics of guideline adherence. Theoretically, this study integrates the four-function model of NSSI and SDT. By clarifying PH as a significant mediator and sex as a key moderator, it elucidates the complex, multidimensional pathways linking movement behaviors to NSSI, thereby validating and extending the 24-h movement framework. Practically, given the notably high prevalence of adolescent NSSI and the relatively low rate of guideline adherence, promoting key movement behaviors, particularly adequate sleep, forms an indispensable component of NSSI prevention strategies. Furthermore, prioritizing PH improvement and accounting for sex differences are critical considerations for targeted interventions.

## Data Availability

The raw data supporting the conclusions of this article will be made available by the authors, without undue reservation.

## References

[1] Nock MK. Self-injury. Annu Rev Clin Psychol. (2010) 6:339–63. doi: 10.1146/annurev.clinpsy.121208.131258 20192787

[2] Xiao Q Song X Huang L Hou D Huang X. Global prevalence and characteristics of non-suicidal self-injury between 2010 and 2021 among a non-clinical sample of adolescents: a meta-analysis. Front Psychiatry. (2022) 13:912441. doi: 10.3389/fpsyt.2022.912441 36032224 PMC9399519

[3] Qu DY Wen X Liu BW Zhang X He YH Chen DY . Non-suicidal self-injury in chinese population: A scoping review of prevalence, method, risk factors and preventive interventions. Lancet Reg Health West Pac. (2023) 37:19. doi: 10.1016/j.lanwpc.2023.100794 37693882 PMC10485683

[4] Daukantaite D Lundh L-G Wångby-Lundh M Claréus B Bjärehed J Zhou Y . What happens to young adults who have engaged in self-injurious behavior as adolescents? A 10-year follow-up. Eur Child Adolesc Psychiatry. (2021) 30:475–92. doi: 10.1007/s00787-020-01533-4 32318877 PMC8019412

[5] Nock MK Joiner TE Gordon KH Lloyd-Richardson E Prinstein MJ. Non-suicidal self-injury among adolescents: diagnostic correlates and relation to suicide attempts. Psychiatry Res. (2006) 144:65–72. doi: 10.1016/j.psychres.2006.05.010 16887199

[6] Klonsky ED May AM Glenn CR. The relationship between nonsuicidal self-injury and attempted suicide: converging evidence from four samples. J Abnorm Psychol. (2013) 122:231–37. doi: 10.1037/a0030278 23067259

[7] Hamza CA Stewart SL Willoughby T. Examining the link between nonsuicidal self-injury and suicidal behavior: a review of the literature and an integrated model. Clin Psychol Rev. (2012) 32:482–95. doi: 10.1016/j.cpr.2012.05.003 22717336

[8] Wang YJ Li X Ng CH Xu DW Hu S Yuan TF. Risk factors for non-suicidal self-injury (nssi) in adolescents: A meta-analysis. EClinicalMedicine. (2022) 46:101350. doi: 10.1016/j.eclinm.2022.101350 35330803 PMC8938878

[9] Moloney F Amini J Sinyor M Schaffer A Lanctôt KL Mitchell RHB. Sex differences in the global prevalence of nonsuicidal self-injury in adolescents: a meta-analysis. JAMA Netw Open. (2024) 7:e2415436. doi: 10.1001/jamanetworkopen.2024.15436 38874927 PMC11179134

[10] Khazaie H Najafi F Chehri A Rahimi-Movaghar A Amin-Esmaeili M Moradinazar M . Physical activity patterns, circadian rhythms, and aggressive and suicidal behavior among a larger sample of the general population aged 15 to 34 years. J Clin Med. (2023) 12:2821. doi: 10.3390/jcm12082821 37109158 PMC10141705

[11] Lai W Wu H Yang L Chen R Xin Z Zhang X . Prevalence of unhealthy behaviors and their associations with non-suicidal self-injury, suicidal ideation and suicide attempt among chinese adolescents. Child Adolesc Psychiatry Ment Health. (2024) 18:61. doi: 10.1186/s13034-024-00742-y 38812024 PMC11137955

[12] Goñi-Sarriés A Gutiérrez-Valencia M Morata-Sampaio L Saiz-Fernández LC Leache-Alegría L Sánchez-Villegas A. Lifestyle habits, problem behaviors and non-suicidal self-injury in adolescents: a systematic review with meta-analysis of longitudinal studies. Adolesc Res Rev. (2026) 11:95–110. doi: 10.1007/s40894-025-00257-3

[13] Huang X Chen Y Luo J Wang D Yang C Luo W . The effect of behavioral activation play therapy in adolescents with depression: a study protocol for a randomized controlled trial. PLoS ONE. (2024) 19:e0304084. doi: 10.1371/journal.pone.0304084 38900751 PMC11189190

[14] Ali A Azam M Mattiullah J. Physical exercise as medicine for self-injurious behavior. Psychiatr Ann. (2020) 50:167–77. doi: 10.3928/00485713-20200302-02

[15] Guo Y Yin X Xu J Chen F Zhang F Liu Y . The relationship between sedentary behavior and non-suicidal self-injury behavior among adolescents in china. Front Psychiatry. (2024) 15:1489707. doi: 10.3389/fpsyt.2024.1489707 39698214 PMC11652529

[16] Vancampfort D Stubbs B Mugisha J Firth J Van Damme T Smith L . Leisure-time sedentary behavior and suicide attempt among 126,392 adolescents in 43 countries. J Affect Disord. (2019) 250:346–53. doi: 10.1016/j.jad.2019.03.053 30877857

[17] Tang Y Wan YH Xu SJ Zhang SC Hao JH Tao FB. Nonlinear relationship between sleep duration and non-suicidal self-injurious behaviour among chinese adolescents. BMC Psychiatry. (2021) 21:14. doi: 10.1186/s12888-021-03539-x 34674680 PMC8532314

[18] Liu XC Liu ZZ Chen RH Cheng XZ Bo QG Wang ZY . Nightmares are associated with future suicide attempt and non-suicidal self-injury in adolescents. J Clinic Psychiatry. (2019) 80:14. doi: 10.4088/JCP.18m12181 31141321

[19] Pedišić Ž. Measurement issues and poor adjustments for physical activity and sleep undermine sedentary behaviour research—the focus should shift to the balance between sleep, sedentary behaviour, standing and activity. Kinesiology. (2014) 46:135–46.

[20] Pedišić Ž Dumuid D. S Olds T. Integrating sleep, sedentary behaviour, and physical activity research in the emerging field of time-use epidemiology: definitions, concepts, statistical methods, theoretical framework, and future directions. Kinesiology. (2017) 49:252–69.

[21] Tremblay MS Carson V Chaput JP Gorber SC Dinh T Duggan M . Canadian 24-hour movement guidelines for children and youth: an integration of physical activity, sedentary behaviour, and sleep. Appl Physiol Nutr Metab. (2016) 41:S311-s27. doi: 10.1139/apnm-2016-0151 27306437

[22] Chaput JP Carson V Gray CE Tremblay MS. Importance of all movement behaviors in a 24 hour period for overall health. Int J Environ Res Public Health. (2014) 11:12575–81. doi: 10.3390/ijerph111212575 25485978 PMC4276632

[23] Rollo S Antsygina O Tremblay MS. The whole day matters: understanding 24-hour movement guideline adherence and relationships with health indicators across the lifespan. J Sport Health Sci. (2020) 9:493–510. doi: 10.1016/j.jshs.2020.07.004 32711156 PMC7749249

[24] Chaput JP Willumsen J Bull F Chou R Ekelund U Firth J . 2020 who guidelines on physical activity and sedentary behaviour for children and adolescents aged 5-17years: Summary of the evidence. Int J Behav Nutr Physic Act. (2020) 17:9. doi: 10.1186/s12966-020-01037-z 33239009 PMC7691077

[25] Huang J Memon AR Bao R Fan H Wang L Liu Y . 24-h movement behaviours research in chinese population: a scoping review. J Exerc Sci Fit. (2024) 22:397–405. doi: 10.1016/j.jesf.2024.07.005 39219863 PMC11363828

[26] Bao R Yang Z Memon AR Chen S Wang L Cai Y. Association between meeting the 24-h movement guidelines and psychosocial health in children: a cross-sectional study. Child Care Health Dev. (2024) 50:e13191. doi: 10.1111/cch.13191 37899718

[27] Hua M Hua Y Peng Y Zhu J. Associations between adherence to 24-hour movement guidelines with depression, anxiety, and loneliness among chinese adolescents. J Affect Disord. (2025) 385:119369. doi: 10.1016/j.jad.2025.05.029 40339715

[28] Tapia-Serrano MA Sevil-Serrano J Sánchez-Miguel PA López-Gil JF Tremblay MS García-Hermoso A. Prevalence of meeting 24-hour movement guidelines from pre-school to adolescence: a systematic review and meta-analysis including 387,437 participants and 23 countries. J Sport Health Sci. (2022) 11:427–37. doi: 10.1016/j.jshs.2022.01.005 35066216 PMC9338333

[29] Sampasa-Kanyinga H Chaput JP Goldfield GS Janssen I Wang J Hamilton HA . 24-hour movement guidelines and suicidality among adolescents. J Affect Disord. (2020) 274:372–80. doi: 10.1016/j.jad.2020.05.096 32469829

[30] López-Gil JF Firth J García-Hermoso A. Is meeting with the 24-h movement recommendations linked with suicidality? Results from a nationwide sample of 44,734 us adolescents. J Affect Disord. (2024) 349:617–24. doi: 10.1016/j.jad.2024.01.019 38190855

[31] Liu BP Jia CX Li SX. The association of soft drink consumption and the 24-hour movement guidelines with suicidality among adolescents of the united states. Nutrients. (2022) 14:1870. doi: 10.3390/nu14091870 35565838 PMC9100874

[32] Freeman M. The world mental health report: transforming mental health for all. World Psychiatry. (2022) 21:391–92. doi: 10.1002/wps.21018 36073688 PMC9453907

[33] Nock MK Prinstein MJ. A functional approach to the assessment of self-mutilative behavior. J Consult Clin Psychol. (2004) 72:885–90. doi: 10.1037/0022-006X.72.5.885 15482046

[34] Nock MK. Why do people hurt themselves?: New insights into the nature and functions of self-injury. Curr Dir Psychol Sci. (2009) 18:78–83. doi: 10.1111/j.1467-8721.2009.01613.x 20161092 PMC2744421

[35] Nock MK Prinstein MJ. Contextual features and behavioral functions of self-mutilation among adolescents. J Abnorm Psychol. (2005) 114:140–46. doi: 10.1037/0021-843X.114.1.140 15709820

[36] Vansteenkiste M Ryan RM. On psychological growth and vulnerability: basic psychological need satisfaction and need frustration as a unifying principle. J Psychother Integr. (2013) 23:263–80. doi: 10.1037/a0032359

[37] Luis SE. Bullying victimization and suicide among us high school adolescents: The mediating role of depressive symptoms and the moderating effects of physical activity and sex. J School Violence. (2025) 1–14. doi: 10.1080/15388220.2025.2596991 [Epub ahead of print].

[38] Wilhite K Booker B Huang BH Antczak D Corbett L Parker P . Combinations of physical activity, sedentary behavior, and sleep duration and their associations with physical, psychological, and educational outcomes in children and adolescents: A systematic review. Am J Epidemiol. (2023) 192:665–79. doi: 10.1093/aje/kwac212 36516992 PMC10089066

[39] Sampasa-Kanyinga H Colman I Goldfield GS Janssen I Wang JL Podinic I . Combinations of physical activity, sedentary time, and sleep duration and their associations with depressive symptoms and other mental health problems in children and adolescents: a systematic review. Int J Behav Nutr Physic Act. (2020) 17:16. doi: 10.1186/s12966-020-00976-x 32503638 PMC7273653

[40] Brown RC Plener PL. Non-suicidal self-injury in adolescence. Curr Psychiatry Rep. (2017) 19:20. doi: 10.1007/s11920-017-0767-9 28315191 PMC5357256

[41] Chen ST Liu Y Tremblay MS Hong JT Tang Y Cao ZB . Meeting 24-h movement guidelines: prevalence, correlates, and the relationships with overweight and obesity among chinese children and adolescents. J Sport Health Sci. (2021) 10:349–59. doi: 10.1016/j.jshs.2020.07.002 32679341 PMC8167320

[42] Liu Y Wang M Tynjälä J Lv Y Villberg J Zhang Z . Test-retest reliability of selected items of health behaviour in school-aged children (hbsc) survey questionnaire in beijing, china. BMC Med Res Methodol. (2010) 10:73. doi: 10.1186/1471-2288-10-73 20696078 PMC2927607

[43] Nupponen H Laakso L Rimpelä A Pere L Telama R. Questionnaire-assessed moderate to vigorous physical activity of the finnish youth in 1979-2005. Scand J Med Sci Sports. (2010) 20:e20–6. doi: 10.1111/j.1600-0838.2009.00875.x 19422649

[44] Buysse DJ Reynolds CF Monk TH Berman SR Kupfer DJ. The pittsburgh sleep quality index - a new instrument for psychiatric practice and research. Psychiatry Res. (1989) 28:193–213. doi: 10.1016/0165-1781(89)90047-4 2748771

[45] Ho KY Lam KKW Xia W Chung JOK Cheung AT Ho LLK . Psychometric properties of the chinese version of the pittsburgh sleep quality index (psqi) among hong kong chinese childhood cancer survivors. Health Qual Life Outcomes. (2021) 19:176. doi: 10.1186/s12955-021-01803-y 34229705 PMC8261921

[46] Yang Y Yuan S Liu Q Li F Dong Y Dong B . Meeting 24-hour movement and dietary guidelines: prevalence, correlates and association with weight status among children and adolescents: a national cross-sectional study in china. Nutrients. (2022) 14:2822. doi: 10.3390/nu14142822 35889779 PMC9317649

[47] Qian H Tan J Tang J Zou Y. Ethnic and residential differences in adherence to the 24-h movement guidelines and self-reported physical fitness among zhuang and han adolescents in southern china. Front Public Health. (2025) 13:1631277. doi: 10.3389/fpubh.2025.1631277 41426690 PMC12711711

[48] Yuhui W Wan L Jiahu H Fangbiao T. Development and evaluation on reliability and validity of adolescent non-suicidal self-injury assessment questionnaire. Chin J School Health. (2018) 39:170–73. doi: 10.16835/j.cnki.1000-9817.2018.02.005

[49] Gao Y Lu C Zhang X Han B Hu H. Physical activity and non-suicidal self-injurious behavior in chinese adolescents: the chain mediating role of psychological capital and relative deprivation. Front Psychiatry. (2024) 15:1509967. doi: 10.3389/fpsyt.2024.1509967 39676912 PMC11638173

[50] Jiang Y Yu H Zheng Q Zhu Y Qin Q Zhang J . Effects of decision making and impulsivity on the addictive features of non-suicidal self-injury behaviors in adolescents with depressive disorder. BMC Psychiatry. (2024) 24:708. doi: 10.1186/s12888-024-06121-3 39425107 PMC11490135

[51] American Psychiatric Association D-TF. Diagnostic and statistical manual of mental disorders: Dsm-5. Washington, DC: American Psychiatric Association Publishing (2013). p. 923-24. doi: 10.1176/appi.books.9780890425596

[52] Wang L Zou H-O Qu Y-H Hong J-F Chen J. The role of pain and blood simulation of nonsuicidal self-injury in emotion regulation among adolescents with depression based on the principle of harm reduction: an experimental study. Behav Ther. (2025) 56:543–54. doi: 10.1016/j.beth.2024.06.002 40287182

[53] Ding D Fang S. Adolescents' psychological flexibility and non-suicidal self-injury: Exploring between-person and within-person association. J Context Behav Sci. (2025) 37:100916. doi: 10.1016/j.jcbs.2025.100916

[54] TAO S WAN Y WU X SUN Y XU S ZHANG S . Evaluation and application of brief instrument on psychological health of youths. Chin J School Health (2020) 41:1331-34+38. doi: 10.16835/j.cnki.1000-9817.2020.09.014

[55] Wang J Xie Y Xu H Wan Y Tao F. Moderating effects of smoking and drinking on the relationship between biological rhythm and psychological health and gender differences among adolescents. BMC Psychiatry. (2023) 23:731. doi: 10.1186/s12888-023-05253-2 37817125 PMC10566120

[56] Zhang Y Xiong J Sun R Chai G Xiong L. Sugar-sweetened beverages, relative grip strength, and psychological symptoms among rural adolescents in western china: A cross-sectional study. Front Nutr. (2025) 12:1511256. doi: 10.3389/fnut.2025.1511256 39886545 PMC11774743

[57] Hayes AF. Introduction to mediation, moderation, and conditional process analysis: A regression-based approach. 3rd ed. New York, NY: The Guilford Press (2022). p. 90–107; 33–36.

[58] Little RJ. A test of missing completely at random for multivariate data with missing values. J Am Stat Assoc. (1988) 83:1198–202. doi: 10.1080/01621459.1988.10478722

[59] Buuren Sv. Flexible imputation of missing data. 2nd ed. New York, NY: Chapman and Hall/CRC (2018). p. 49.

[60] Rubin DB. Multiple imputation for nonresponse in surveys. New York, NY: Wiley & Sons, Inc (1987). p. 66–67. doi: 10.1002/9780470316696

[61] Sterne JA White IR Carlin JB Spratt M Royston P Kenward MG . Multiple imputation for missing data in epidemiological and clinical research: Potential and pitfalls. BMJ. (2009) 338:b2393. doi: 10.1136/bmj.b2393 19564179 PMC2714692

[62] Podsakoff PM MacKenzie SB Lee JY Podsakoff NP. Common method biases in behavioral research: a critical review of the literature and recommended remedies. J Appl Psychol. (2003) 88:879–903. doi: 10.1037/0021-9010.88.5.879 14516251

[63] Cai S Zhong P Dang J Liu Y Shi D Chen Z . Associations between combinations of 24-h movement behaviors and physical fitness among chinese adolescents: sex and age disparities. Scand J Med Sci Sports. (2023) 33:1779–91. doi: 10.1111/sms.14427 37309995

[64] Guthold R Stevens GA Riley LM Bull FC. Worldwide trends in insufficient physical activity from 2001 to 2016: a pooled analysis of 358 population-based surveys with 1·9 million participants. Lancet Global Health. (2018) 6:e1077–e86. doi: 10.1016/S2214-109X(18)30357-7 30193830

[65] Liu L Liu Y Liu Z Zhao L Zhang X Tang P . Associations between loneliness and psychological health symptoms as well as dietary habits in adolescents: Sex differences. J Affect Disord. (2026) 392:120267. doi: 10.1016/j.jad.2025.120267 40930179

[66] Zhao L Hong S Peng X He X Hu J Ma L . Impaired response inhibition to negative emotional stimuli in depressed adolescents with non-suicidal self-injury: a neurophysiological perspective. Front Psychiatry (2025) 16:9068. doi: 10.3389/fpsyt.2025.1559068 40236493 PMC11996638

[67] Zhu Y Du Z Zhang Y Li X Meng J. A study of the symptom network structure of internet addiction, non-suicidal self-injury and internalization problems in adolescents and its association with physical activity. BMC Public Health. (2025) 25:4065. doi: 10.1186/s12889-025-25370-9 41266999 PMC12632008

[68] Lan Z Pau K Md Yusof H Huang X. The effect of emotion regulation on non-suicidal self-injury among adolescents: the mediating roles of sleep, exercise, and social support. Psychol Res Behav Manag. (2022) 15:1451–63. doi: 10.2147/PRBM.S363433 35698565 PMC9188465

[69] Lee HS Rhee SJ Kang DH Min S Hong M Lee H . Non-suicidal self-injury and exercise: associations with addiction. J Korean Med Sci. (2025) 40:e176. doi: 10.3346/jkms.2025.40.e176 40762593 PMC12322591

[70] Boone SD Brausch AM. Physical activity, exercise motivations, depression, and nonsuicidal self-injury in youth. Suicide Life Threat Behav. (2016) 46:625–33. doi: 10.1111/sltb.12240 26970091

[71] Yu H Mu Q Li K. Effects of physical exercise on non-suicidal self-injury in adolescents: the chain mediating role of perceived social support and self-concept. Front Psychiatry. (2023) 14:1201863. doi: 10.3389/fpsyt.2023.1201863 37876620 PMC10591106

[72] Tremblay MS. Introducing 24-hour movement guidelines for the early years: a new paradigm gaining momentum. J Phys Act Health. (2020) 17:92–5. doi: 10.1123/jpah.2019-0401 31711035

[73] Marciano L Ostroumova M Schulz PJ Camerini AL. Digital media use and adolescents' mental health during the covid-19 pandemic: a systematic review and meta-analysis. Front Public Health. (2022) 9:793868. doi: 10.3389/fpubh.2021.793868 35186872 PMC8848548

[74] Goldstein AN Walker MP. The role of sleep in emotional brain function. Annu Rev Clin Psychol. (2014) 10:679–708. doi: 10.1146/annurev-clinpsy-032813-153716 24499013 PMC4286245

[75] Ranum BM Wichstrøm L Pallesen S Falch-Madsen J Halse M Steinsbekk S. Association between objectively measured sleep duration and symptoms of psychiatric disorders in middle childhood. JAMA Netw Open. (2019) 2:e1918281. doi: 10.1001/jamanetworkopen.2019.18281 31880797 PMC6991225

